# Toward a Smartphone Application for Estimation of Pulse Transit Time

**DOI:** 10.3390/s151027303

**Published:** 2015-10-27

**Authors:** He Liu, Kamen Ivanov, Yadong Wang, Lei Wang

**Affiliations:** 1 The Shenzhen Institutes of Advanced Technology, Chinese Academy of Sciences, Shenzhen 518055, China; E-Mails: he.liu.doc@gmail.com (H.L.); kamen@siat.ac.cn (K.I.); 2 The Biomedical Engineering Department, Harbin Institute of Technology, Harbin150001, China

**Keywords:** pulse transit time (PTT), photoplethysmographic imaging (PPGi), microcamera, smartphone, signal processing

## Abstract

Pulse transit time (PTT) is an important physiological parameter that directly correlates with the elasticity and compliance of vascular walls and variations in blood pressure. This paper presents a PTT estimation method based on photoplethysmographic imaging (PPGi). The method utilizes two opposing cameras for simultaneous acquisition of PPGi waveform signals from the index fingertip and the forehead temple. An algorithm for the detection of maxima and minima in PPGi signals was developed, which includes technology for interpolation of the real positions of these points. We compared our PTT measurements with those obtained from the current methodological standards. Statistical results indicate that the PTT measured by our proposed method exhibits a good correlation with the established method. The proposed method is especially suitable for implementation in dual-camera-smartphones, which could facilitate PTT measurement among populations affected by cardiac complications.

## 1. Introduction

Pulse transit time (PTT) is the time required for a pulse wave to travel from one arterial site to another, and it can serve as a measure of the mean pulse wave. It is well known that PTT is highly correlated with the elasticity, compliance, and rigidity of vascular walls [1], left ventricular ejection time [2], and blood pressure variations [3,4]. PTT has also demonstrated its potential as a noninvasive surrogate marker for respiratory conditions, such as upper airway resistance syndrome [5,6], obstructive sleep apnea syndrome [7,8], sleep respiratory events and microarousals in children [9,10].

The conventional method of acquiring PTT combines an electrocardiographic (ECG) signal and a photoplethysmographic (PPG) signal, *i.e*., ECG-PPG principle [11]. However, the ECG part of this technique requires the use of at least three wet adhesive Ag/AgCl ECG electrodes. The long time monitoring using such electrodes, and their cost appear as disadvantages of the ECG-PPG technique. Apart from the ECG-PPG principle, it is possible to measure PTT using two pulse wave signals captured from two different arterial sites, *i.e*., using a PPG-PPG technique. Recently, many PPG-PPG methods for measurement of PTT have been proposed. Campo *et al.* have introduced a method based on laser Doppler vibrometry to measure PTT on the skin surface of the common carotid artery [1]. Hahn *et al.* suggested a novel method to estimate the aortic-to-peripheral PTT using blood pressure signals captured from two diametric peripheral locations [12]. Xu *et al.* presented a method based on system identification analysis to improve PTT estimation precision [13]. Rashedi *et al.* introduced two alternative tube-load models that could enable estimation of the relationship between PTT and peripheral arterial blood pressure waveforms [14].

Alternatively, photoplethysmographic imaging (PPGi), using microcamera(s) to detect PTT, is a less complicated method that is also noninvasive [15,16]. Chandrasekaran *et al.* developed a new application of the smartphone using its camera and Cardiechema-PPGi technology to acquire PTT [17]. However, the application requires a microphone connected to the smartphone in place of a stethoscope. 

Smartphones that are equipped with two opposing microcameras are ubiquitous among the general public. However, their potential for extracting physiological parameters from simultaneously recorded PPGi signals has not been explored. As described in existing literature, PPG signals may be subjected to subsequent PTT analysis, and the time interval between PPG signals from the same pulse pressure wave at different arterial sites is highly correlated with the PTT [18]. This paper presents a method for PTT estimation that uses two opposing cameras, acquiring PPGi signals simultaneously, one from the index fingertip and the other from the skin surface at the forehead temple. This paper also presents the algorithm that was developed for the detection of maxima and minima in the PPGi waveform signals. It allows for effectively distinguishing the incident wave maxima from reflected wave ones. This was achieved by utilizing a quadratic function. Another two quadratic functions were employed to enhance the temporal accuracy in determining the locations of maxima and minima respectively, thus to improve the accuracy of PTT measurement. Finally, the PTT data acquired with the proposed PPGi method is compared with data obtained by the established ECG-PPG method.

## 2. System Components and Configuration

### 2.1. Image Sensor Module

Two identical Ov9715 digital cameras, available from OmniVision for less than $10 each, were used as image sensors. The Ov9715 is a low voltage and high-performance CMOS wide extended graphics array (WXGA) camera (1280 × 800 pixels), operating at a fixed rate of 30 frames per second (fps), with all functional units integrated on a single chip. These cameras provide full-frame and cropped 8-bit or 10-bit images in unprocessed red/green/blue (RGB) format through a digital video port (DVP).

### 2.2. Configuration of Analytic System

**Figure 1:**
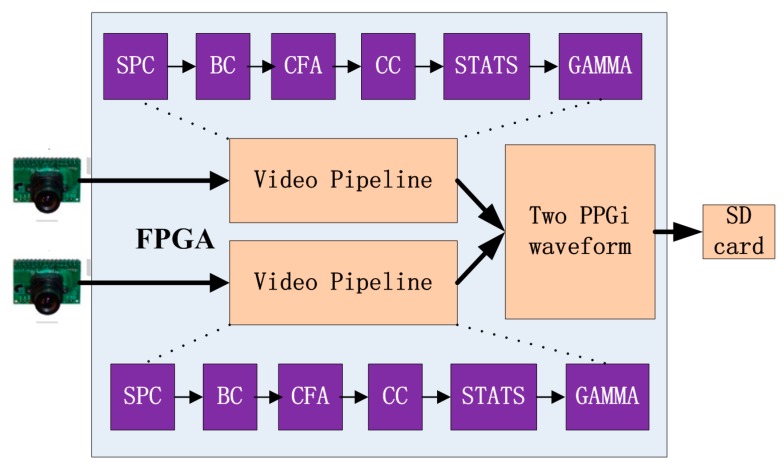
Acquisition system used to obtain the two PPGi waveforms, based on a field programmable gate array (FPGA) development board. The system includes a stuck pixel correction unit (SPC), brightness and contrast control unit (BC), Bayer conversion unit (CFA), color balance control (CC), image statistics unit (STATS), and gamma correction unit (GAMMA).

The hardware used for this study was based on our previous work [19,20]. The system consists of the two commercial cameras described previously connected to a field programmable gate array (FPGA) development board (XC6SLX150T-3FGG676 from Xilinx), in turn connected to a secure digital (SD) card (Figure 1). All features of the cameras, including the exposure control, white balance control, defective pixel correction, and others, were configured by commands sent from the development board to the cameras through the serial camera control buses. Proper adjustment of the camera settings produced clear images. In the development board, data from the two cameras were first passed to the video pipeline and then the extracted PPGi waveform data were recorded in a text file on the SD card. This text file was copied to a PC for subsequent data analysis.

### 2.3. Region of Interest (ROI)

Several skin surface locations are suitable for acquiring PPGi waveform signals, such as the fingertips, toes, earlobes, and temple [21]. The blood perfusion levels in the regions of fingers, palms, face, and ears are much higher than those of all other body locations [22]. Since the length of the arterial path between two sites is critical for the accuracy of PTT measurement [23], we selected the right fingertip and the forehead temple as ROIs to ensure the optimal distance between the two sites and facilitate accurate measurements.

### 2.4. Experimental Protocol

A total of twelve healthy volunteers (ten males and two females, ages 24 to 35, with a mean age of 28.6 years) were recruited from the Shenzhen Institutes of Advanced Technology, Chinese Academy of Sciences. To investigate the accuracy of the PTT estimation, Experiment 1 measured the time interval between a minimum in the PPGi waveform signal from one location and the subsequent minimum in the PPGi waveform signal from the second location. This measurement served as a reference for Experiment 2. Experiment 2 measured the PTT as a difference between a minimum in the PPGi waveform signal from the temple and the subsequent maximum in the PPGi waveform signal from the fingertip within the same cardiac cycle. We investigated the correlation of this PTT with the PTT obtained using the established ECG–PPG method.

#### 2.4.1. Experiment 1 Setup

**Figure 2:**
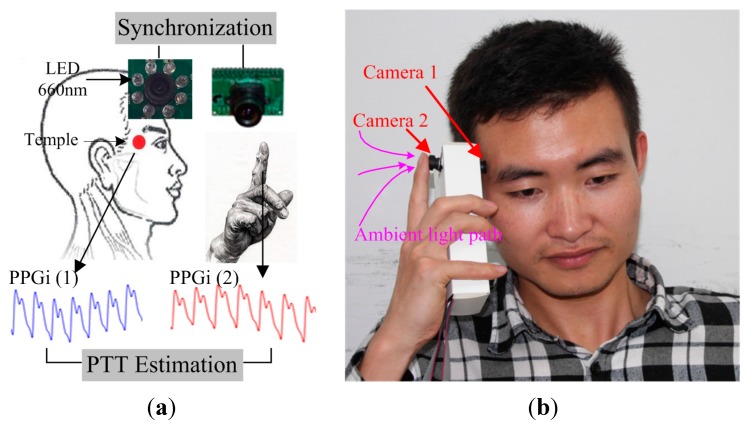
Experimental design for PTT estimation: (**a**) Schematic diagram showing two PPGi signals recorded simultaneously. The first camera obtained Signal (1) from the skin surface of the temple. The second camera obtained Signal (2) when the fingertip was placed over the lens; (**b**) A proposed design that would utilize the opposing cameras in smartphones.

The reference PTT was obtained using two PPG modules (both TP-TSD200A from BIOPAC). One PPG signal was captured from the index fingertip of the right hand, and the other PPG signal was captured from the temple. The reference PTT was defined as the time interval between the minimum of the PPG waveform signal in the temple channel and the minimum of the immediately subsequent PPG waveform signal in the fingertip channel. PPG signals were synchronously acquired at a sampling rate of 1 kHz. Each subject was asked to avoid bodily movement and to breathe normally during the image capture. Figure 2a shows a schematic diagram that illustrates the suggested PTT estimation method using the two cameras. The first camera was placed over the temple, with eight 660 nm LEDs attached to the lens for a light source. Simultaneously, the right index fingertip was placed over the lens of one of the cameras, forming the second PPGi waveform, with ambient light as a light source. The high pressure exerted by the hand ensured sufficient contact between the camera lens and the skin surface, resulting in excellent PPGi waveform acquisition quality. The temperature of the fingertips also affects the PPGi signal [24], so a temperature sensor (TSD202D from BIOPAC) was used to record the temperature of the right fingertip at the beginning and the end of the experiments. Figure 2b illustrates the implementation of a prototype design that resembles the opposing cameras in a smartphone. The experiments were conducted indoors and daylight combined with standard artificial fluorescent light served as ambient light.

#### 2.4.2. Experiment 2 Setup

The procedure for Experiment 2 differed from that of Experiment 1 in the selection of the reference PTT. The PTT measurement system in Experiment 2 used one TP-TSD200A PPG module and one TP-TSD155C ECG system, both from BIOPAC. For Experiment 2, standard 1-lead ECG signals were captured by placing three electrodes on the subject, one each on the right wrist, left wrist, and right leg. The PPG signal was captured from the index fingertip of the subject’s right hand. Both the ECG and PPG signals were synchronously acquired at a sampling rate of 1 kHz. The reference PTT was defined as the time interval between a maximum in the ECG R-wave and one of the extreme points of the PPG wave from the fingertip. Consequently, the PTTs for the both cases—the minimum and the maximum of the PPG wave were obtained.

## 3. Algorithm

### 3.1. Image Acquisition and Processing

For each of the cameras, one PPGi waveform was selected from among the red, green, and blue channels to ensure the best signal to noise ratio (SNR). In our previous study [25], we investigated the PPGi signals obtained from the three color channels using light of several different wavelengths for the illumination. The PPGi signal from the red channel showed the best SNR compared to the green and blue channels under illumination light with a wavelength of 660 nm. In other studies [26,27] the PPGi signal from the green channel showed the best SNR compared to the red and blue channels under ambient light illumination. Our present work is consistent with these studies, *i.e*., we used the green channel for the fingertip signal and the red channel for the temple signal. For each channel, 8-bit images were captured at a frame rate of 30 fps. The images were then passed to the FPGA system. The FPGA system acquired the pixel values from the ROI in each frame and then averaged the values. Thus, a temporally sequential series of values was obtained for each camera and synchronously recorded on the SD card. The two series supplied the inputs for offline processing using MATLAB 2013b (MathWorks, Inc., Natick, MA, USA).

### 3.2. Detection of Maxima and Minima in PPGi Signals

**Figure 3:**
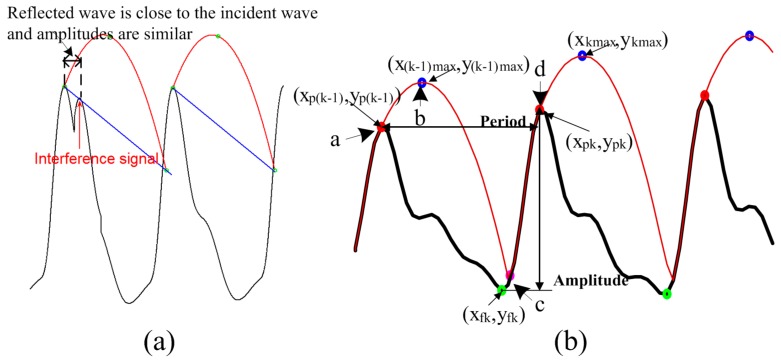
Incident and reflected waves in PPGi signals: (**a**) Limitation of the method in [29] due to interference from reflected waves; (**b**) Illustration of the SFP technique for detection of maxima.

Compared with conventional PPG signals, PPGi signals exhibit significant baseline fluctuation (*i.e*., AC component of less than 0.6 Hz). Furthermore, in some cases, the amplitudes of reflected waves are similar to the amplitudes of their incident waves. In addition, there are sudden changes upwards or downwards in the baseline level of the PPGi wave. These differences from the conventionally acquired signals have two major sources. First, PPGi signals are susceptible to interference from motion, since physical displacements influence photon propagation and hence the effective optical path length [28]. In our experiments, PPGi signals were acquired in an ordinary environment, where the subjects placed their index fingertips on the camera lens with intentional but not excessive force, and differences in light conditions for the two cameras were avoided. Under these conditions, the presence of minor motion artifacts was inevitable, and, combined with the superposition of a quasi-periodic respiration wave, resulted in baseline fluctuations. Second, previous studies [29,30] demonstrated that factors such as aging, hypertension, and arterial sclerosis may result in larger amplitudes of reflected waves and shorter distances between the incident wave and the reflected wave. In our experiments, due to the motion interferences, reflected wave amplitudes were often similar to incident wave amplitudes, and it was difficult to differentiate between the reflected and incident waves (Figure 3a). Although a digital filtering method may remove baseline distortion, severe morphological distortions from low perfusion and baseline drift are hard to eliminate with frequency filtering. An additional filtering or feature extraction method, such as a moving average filter or wavelet decomposition, may facilitate regulation of the signal; however, filtering procedures produce phase shifts, resulting in a time delay. Therefore, the error continues to impact PTT calculation. Many techniques have been suggested to detect the maxima and minima of PPGi signals. However, to the best of our knowledge, none of them is suitable for application to PPGi signals, since none of them takes into account the baseline drift that is typical for PPGi signals. Even more, according to our investigation, the few technologies [29] that were claimed to be applicable for PPGi signals, perform poorly in case of significant baseline drifts.

An adaptive quadratic curve was used to skip the reflected wave peaks in the PPGi signal while detecting the incident wave peaks only. In each cycle, the quadratic curve was determined by the incident wave maximum and the calculated maximum of the quadratic curve. This process is illustrated in Figure 3b. Assume the quadratic curve begins from the maximum of the PPGi signal (point “a”) and arrives at the quadratic curve maximum (point “b”). Then the quadratic curve decreases from its maximum until it reaches the rising stage of the next PPGi signal cycle (point “c”). From there (point “c”), the curve follows the rising stage of the signal until reaching its next maximum point (point “d”), then the process repeats. For each cycle of the PPGi signal, the minimum was determined by choosing the minimum value between the two maximum values on either side of the minimum. Having these values, the maximum of the quadratic curve in each cycle was calculated as follows:

(1) $$\left\{ \begin{array}{l} x_{k\max}=x_{pk}+f_{x}\times (x_{pk}-x_{p(k-1)})\\ y_{k\max}=y_{pk}+f_{y}\times (y_{pk}-y_{fk}) \end{array}\right.$$

where k denotes the cycle number, and $x_{k\max}$ and $y_{k\max}$ locate the maximum of the quadratic curve in the k-th cycle. $x_{p(k-1)}$ and $x_{pk}$ represent the times when the maxima in the (k-1)-th and the k-th cycles were captured, respectively. $y_{fk}$ and $y_{pk}$ represent the amplitudes of the minimum and maximum in the k-th cycle, respectively. $f_{x}$ and $f_{y}$ are coefficients, empirically selected as 0.3 and 0.36, respectively.

With the coordinates for the apex of the quadratic curve in the k-th cycle from Equation (1), this curve is fully determined by

(2) $$\left\{ \begin{array}{l} x_{k\max}=-\frac{b}{2\times a}\\ y_{k\max}=\frac{4\times a\times c-b^{2}}{4\times a\times c}\\ y_{pk}=a\times x_{pk}^{2}+b\times x_{pk}+c \end{array}\right.$$

Repetitions of this process ensured detection of all PPGi signal maxima.

The algorithm was initialized as follows. We wait to detect a segment containing five sequentially rising points in the PPGi signal. When such segment is detected, we consider that its maximum and minimum (*i.e*., the c-d stage in Figure 3b) correspond to the maximum and minimum of the systolic upstroke stage, respectively. Then, we find the maximum and the minimum values of the discovered segment and consider them as the first detected PPGi signal maxima and minima points. Thus, we can acquire $\left(y_{pk}-y_{fk}\right)$. We use 33 sampling points of the signal after the discovered maxima (which corresponds to signal length of 1 s) and perform fast Fourier transform over this signal part. Thus, we obtain the period $\left(x_{pk}-x_{p(k-1)}\right)$. Finally, we calculate the initial quadratic curve according to Equations (1) and (2). The selected number of sequential points ensures that a reflected wave rising stage may not be wrongly detected as the rising stage of an incident wave because the duration of a reflected wave is much shorter than that of an incident wave.

### 3.3. Improving the Detection Accuracy

**Figure 4:**
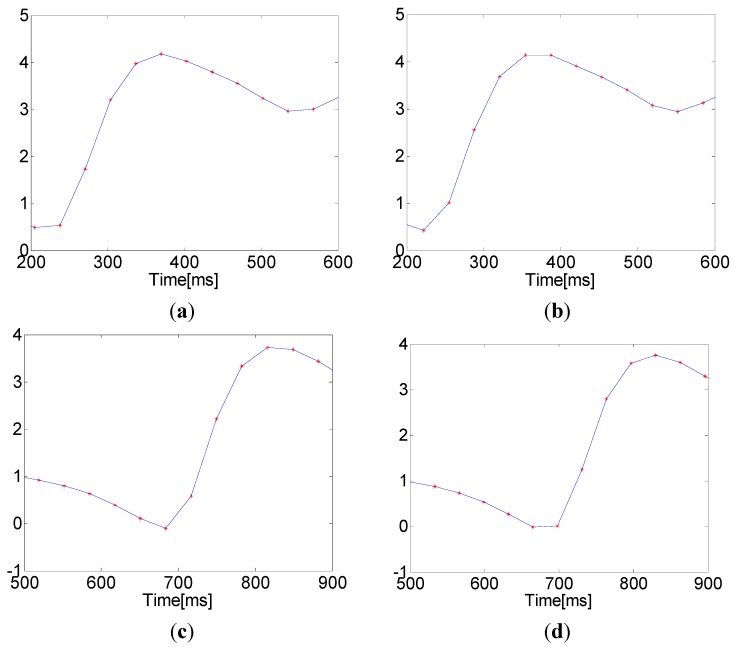
Maxima and minima with rounded and horizontal patterns due to a low sampling frequency: (**a**) a rounded maximum that matches the established ECG-PPG technique; (**b**) a horizontal maximum indicating an error; (**c**) a rounded minimum that matches the established ECG-PPG technique; and (**d**) a horizontal minimum indicating an error.

**Figure 5:**
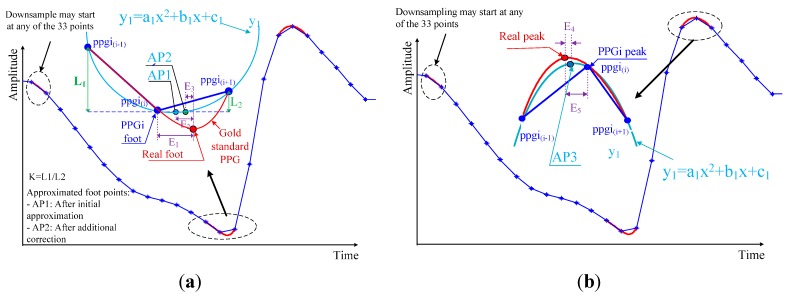
Illustration of minimum (**a**) and maximum (**b**) point detection for the PPGi signal. E_1_ is the error in elapsed time determined by the difference between the minimum of the PPGi signal and the actual minimum indicated by the reference PPG signal. E_2_ is the error after this initial approximation. E_3_ is the difference between AP2 and the actual minimum indicated by the reference PPG signal. E_4_ is the time error determined by the difference between the maximum of the PPGi signal and the actual maximum indicated by the reference PPG signal. E_5_ is the error after this approximation.

The accuracy of the PTT measurement depends entirely upon an accurate determination of maximum and minimum points in the PPGi signal. However, the microcameras’ frame rate of 30 fps is significantly lower than that used in the established ECG-PPG method. Due to this low sampling frequency, the PPGi signal may not accurately represent the maximum and minimum positions. This results in a relatively high error in the measurement of elapsed time, as shown in Figure 4. The algorithm presented here for the approximation of actual maximum and minimum locations is based on the assumption that the minima and maxima in the PPGi signal conform to a parabolic shape, as illustrated in Figure 5. The extremes are considered with the two neighboring points on either side as three points on a parabola (in Figure 5, these are represented by $ppgi_{i}$, $ppgi_{i-1}$, and $ppgi_{i+1}$, respectively). Using these points, we were able to construct the parabola. As illustrated in Figure 5, the extremum of this parabola is the point AP1 in case of a foot, and AP3 in case of a peak. Thus, AP1 and AP3 are the initially approximated points obtained in an attempt to improve the measurement accuracy.

As a result, we achieved the error E_2_, which was lower than E_1_. The reason for the relatively large error E_2_ after the initial approximation is that the curve of the PPGi signal at the minimum is asymmetric, while our curve-fitting function y_1_ is quadratic, and, therefore, symmetric with respect to its vertex. It was also observed that the slope of the PPGi curve after the minimum was steeper than the curve before the minimum, and both slopes related to the actual minimum position indicated by the PPG signal. Based on this observation, to further improve the accuracy, two coefficients, *k_1_* and *k_2_*, were introduced, and their values were determined by the positions of $ppgi_{i-1}$ and $ppgi_{i+1}$ relative to $ppgi_{i}$. *k_1_* adjusts the vertical position of the minimum and was defined as follows:

(3) k1={k1kk≤1k>1, k=|ppgii−ppgii−1ppgii−ppgii+1|

*k_2_* adjusts the horizontal position of the minimum. This coefficient correlates with the period of the PPGi signal and was defined as follows:

(4) k2=hr90

where hr is the period of the PPGi signal.

The point AP2 was defined according to Equation (5). The exponent c is used to minimize E_3_, and its value was determined empirically by a series of statistical tests, indicating the optimal value: c = 1/8.

(5) $$AP2=-\frac{b_{1}}{2\times a_{1}}+k_{1}^{c}\times k_{2}$$

The curve of the PPGi signal at the maximum is more symmetric than at the minimum, so there was no need to apply additional corrections to the initial approximation (AP3, in Figure 5b) to correct the error in the location of the maximum point.

### 3.4. PTT Estimation

**Figure 6:**
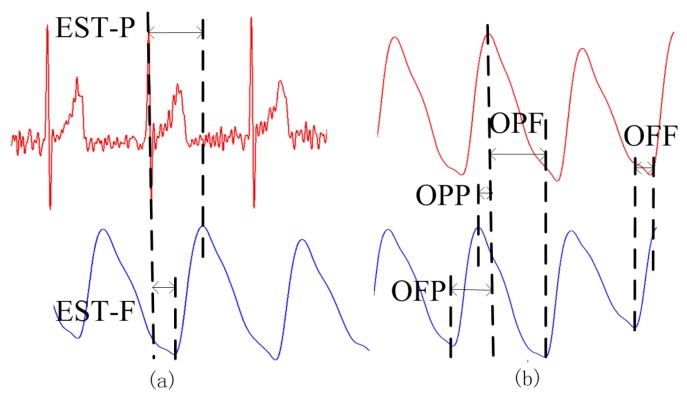
PTT measurements obtained using (**a**) the established ECG-PPG method; and (**b**) the proposed PPGi method.

For the established method of PTT measurement, EST was defined as the time interval between an ECG R-wave maximum and one of the extreme points (maximum or minimum) of the PPG signal from the fingertip. As shown in Figure 6a, EST_P was the time interval between the maximum of the ECG R-wave and the maximum of the PPG signal in the same cardiac cycle. EST_F was the time interval between the maximum of the ECG R-wave and the minimum immediately following the PPG pulse. For our proposed PPGi method, OUR was defined as the time interval between an extreme point (maximum or minimum) from one PPGi signal and the subsequent extreme point (maximum or minimum) from the other PPGi signal in the same cardiac cycle. As shown in Figure 6b, OPP was the time interval between two consecutive maxima, one from each of the PPGi signals. OPF was the time interval between a maximum of the PPGi signal from the fingertip and the subsequent minimum of the PPGi signal from the temple. OFP was the time interval between a minimum of the PPGi signal from the temple and the subsequent maximum of the PPGi signal from the fingertip. OFF was the time interval between two consecutive minima, one from each of the PPGi signals. Each of the time intervals measured by the PPGi method was determined from two points in the same cardiac cycle.

The correlation between PTTs was evaluated for eight pairs, namely EST_P and OPP, EST_F and OPP, EST_P and OPF, EST_F and OPF, EST_P and OFP, EST_F and OFP, EST_P and OFF, and lastly, EST_F and OFF.

### 3.5. Evaluation

Evaluation of the algorithm used to detect signal maxima and minima determines if the PTT obtained by the proposed method is accurate. Sensitivity (SE), Positive predictive value (PPV) were used to evaluate detection algorithm accuracy, respectively. SE is the ability of a test to detect a true positive and it is calculated as shown in Equation (6). Positive predictive value is the proportion of positive test that are true positives and represent the presence and calculated as shown in Equation (7).

(6) $$SE=\frac{TruePositive}{TruePositive+FalseNegative}$$

(7) $$PPV=\frac{TruePositive}{TruePositive+FalsePositive}$$

True positive is correctly identified; False positive is incorrectly identified; False negative is incorrectly rejected.

In addition, statistical analysis was performed using the SPSS software package (version 17.0 from IBM). A Bland-Altman plot [31] compared the PTTs obtained by the proposed PPGi method and the established ECG-PPG method. The mean deviation and standard deviation (SD) of the differences, the mean of the absolute differences were calculated, indicating 95% limits of agreement (±1.96 SD).The correlation between PTTs in several PTT pairs was analyzed using the correlation coefficient *r*; absolute values of *r* > 0.8 indicated highly correlated PTTs. The linear regression was determined using the least squares method.

## 4. Results

The skin temperature at the measurement site was measured at the beginning and at the end of the experiment in the form of mean ± SD. At the beginning and end of the experiment, finger temperatures were 29.8 ± 2.0 °C and 30.4 ± 2.1 °C, respectively. These measurements indicate that during the experiment, there was no significant change in the skin temperature at the measurement site (*p* = 0.81). There was practically no difference in temperatures at the beginning and the end of the experiment.

### 4.1. Performance in Detection of Maxima and Minima

**Figure 7:**
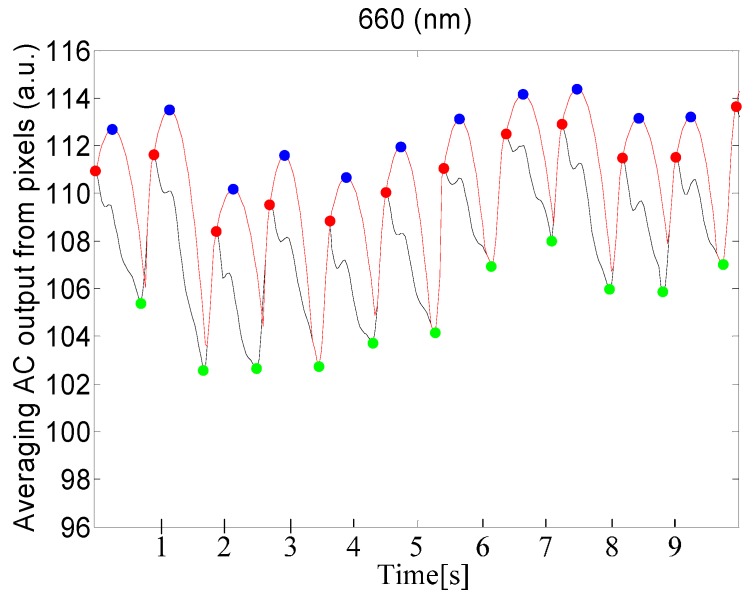
Results of detection of maxima and minima for PPGi signal, using a 660 nm source of light. The red curve represents the adaptive quadratic curve generated by the proposed method. The blue points represent the maxima of the quadratic curve. The red points represent the maxima of the PPGi signal. The green points represent the minima in the PPGi signal. All of these points were determined using the proposed PPGi method.

Table 1 shows statistical results for the performance of maxima and minima detection and provides a comparison between our proposed method and the Shin's method. For our method, SE and PPV of the minima detection are better than that of the maxima detection. In addition, our proposed method shows better performance than the Shin’s method. Figure 7 shows the detected maxima and minima for PPGi signal using the proposed method.

**Table 1:** Maxima and minima detection result.

Illumination Light (Site)	Method	Maximum		Minimum	
		SE	PPV	SE	PPV
660 nm (temple)	Shin’s method	76.72%	88.24%	88.24%	99.98%
	The proposed method	92.85%	97.44%	97.44%	100%
Ambient light (fingertip)	Shin’s method	89.27%	97.72%	97.72%	100%
	The proposed method	98.07%	98.64%	98.64%	100%

### 4.2. Performance of Corrective Adjustments to Locations

The errors in detection of maxima and minima in unprocessed PPGi signals after applying the correction in Equations (3)–(5) were expressed in the form of mean bias d ± SD. The error in detection of maxima in the unprocessed PPGi signals was d = −0.39 ms, SD = ±9.76 ms. After applying the correction, the mean bias became d = −0.80 ms, SD = ±1.15 ms. The error in detection of minima in the unprocessed PPGi signals was d = 2.00 ms, SD = ±9.67 ms, becoming d = −1.58 ms, SD = ±1.01 ms after applying the correction. These calculated values for the error in detection of maxima and minima with and without correction were compared with the corresponding errors in the values obtained by the established ECG–PPG method.

### 4.3. PTT Acquisition and Evaluation

#### 4.3.1. Experiment 1

**Figure 8:**
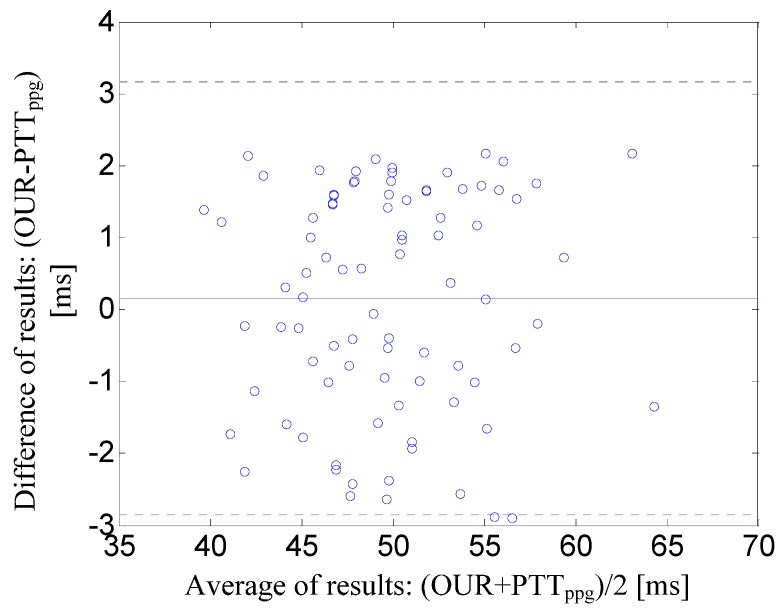
Bland-Altman plot of the PTT derived from the time interval between two PPGi signals (*i.e*., OFF) *versus* the PTT derived by the established ECG-PPG method from the time interval between ECG and PPG signals. The solid line represents the mean bias. Dotted lines represent the limits of agreement within 95% (±1.96SD).

Figure 8 illustrates the Bland-Altman plot of the PTT derived from the time interval between two PPGi signals (*i.e*., the time interval OFF) *versus* the PTT derived by the established ECG–PPG method from the time interval between ECG and PPG signals. Figure 8 shows that the mean bias was d = 0.19 ms with limits of agreement within 95% from −1.34 to 1.72 ms.

#### 4.3.2. Experiment 2

Table 2 provides a summary of correlation coefficients, expressed in absolute value, for each pair of PTTs in twelve subjects. The pair OFP and EST_P showed the highest correlation (0.86 ± 0.06). Therefore, the analysis that follows will concentrate on the relationship between OFP and EST_P, since it shows the most potential for practical application.

**Table 2:** Correlation coefficients (*r*) comparing pulse transit time (PTTs) from established and proposed methods, and direction of linear regression.

	|r| (mean ± SD)	Regression Slope	|r|≥0.6
EST_P~OPP	0.65 ± 0.14	Negative	8
EST_F~OPP	0.05 ± 0.18	N/A ^a^	0
EST_P~OPF	0.68 ± 0.07	Negative	11
EST_F~OPF	0.23 ± 0.11	Positive	0
EST_P~OFP	0.86 ± 0.06	Positive	12
EST_F~OFP	0.60 ± 0.09	Positive	5
EST_P~OFF	0.57 ± 0.15	Positive	4
EST_F~OFF	0.76 ± 0.12	Positive	11

^a^ N/A indicates no consistent direction.

**Figure 9:**
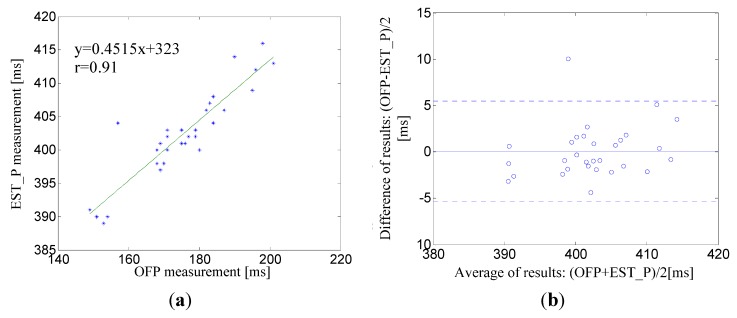
(**a**) A correlation analysis and linear regression line for the pair EST_P–OFP for a male subject, age 30; (**b**) The corresponding Bland-Altman plot between EST_P and linear regression line. The solid line represents the mean of differences. Dotted lines represent the upper and lower limits of agreement, *i.e*., +1.96SD and −1.96SD, respectively. SD is the standard deviation.

For OFP and EST_P the slope of the linear regression was always positive, indicating that OFP is directly proportional to EST_P. Figure 9a shows the linear regression and correlation coefficient (r = 0.91) for the pair OFP and EST_P for a male subject, age 30. The corresponding Bland-Altman plot between EST_P and the linear regression shows that the mean bias was d = 0.08 ms with limits of agreement within 95% from −5.34 to 5.5 ms whose maximum error was less than 2.5% (Figure 9b).

## 5. Discussion

### 5.1. PTT Detection

EST was the time interval between an ECG R-wave and the maximum (EST_P) or minimum (EST_F) points of the PPG signal. In a previous work, it was reported that the values of EST_P and EST_F depended on the physical conditions of particular patients, such as blood pressure, age, or others [4]. OUR was the time interval between and extreme point in one PPGi signal and the subsequent extreme point in the other PPGi signal. Nevertheless, OUR differed from the EST due to the different methods of measurement used. Eight pairs of OUR were compared to EST to identify correlations between the two methods. This analysis demonstrated that OFP showed the highest correlation with EST_P, with deviations of d = −0.01 ms and SD = ±2.82 ms between EST_P and the linear regression of OFP.

EST was the time interval between an ECG R-wave and the maximum (EST_P) or minimum (EST_F) points of the PPG signal. In a previous work, it was reported that the values of EST_P and EST_F depended on the physical conditions of particular patients, such as blood pressure, age, or others [4]. OUR was the time interval between and extreme point in one PPGi signal and the subsequent extreme point in the other PPGi signal. Nevertheless, OUR differed from the EST due to the different methods of measurement used. Eight pairs of OUR were compared to EST to identify correlations between the two methods. This analysis demonstrated that OFP showed the highest correlation with EST_P, with deviations of d = −0.01 ms and SD = ±2.82 ms between EST_P and the linear regression of OFP.

The method proposed here for estimating PTT includes detection of maxima and minima, combined with additional processing to improve accuracy. A previous method described in [29] shows excellent performance in detection of maxima and minima in PPGi signals. However, it is limited because PPGi signals contain a great deal of motion artifacts and considerable baseline fluctuations. To overcome these, we proposed the method described here for detection of maxima and minima in PPGi signals.

The sampling rate of the camera was approximately 30 Hz, allowing time resolution of approximately 33 ms. However, this sampling rate leads to unacceptable errors, since OUR is in the range from 35 ms to 220 ms. To address this problem and improve the detection accuracy, we applied a special corrective algorithm as illustrated in Figure 5. As shown in Figure 8, the mean bias was d = 0.19 ms, with limits of agreement within 95% from −1.34 to 1.72 ms.

The light sources for the cameras imaging at the fingertip and the temple are different. The ambient light is sufficient for the camera imaging at the fingertip [16]. An additional light source, such as the 660 nm wavelength light used for this analysis, is necessary for acquisition of the PPGi signal from the temple because the skin and tissue are thicker at the temple than at the fingertip, and the amplitude of the PPGi signal from the temple is weaker. 

It was accepted in the literature to search for correlation between EST_F and OFF, as well as between EST_F and OPP even though the absolute value of the correlation coefficient of these pairs is lower than that of the pair EST_P—OFP. In our results, for a number of cases the correlation coefficients for the pairs EST_F—OFF and EST_F—OPP exceeded 0.8 which proves the satisfactory performance of our method. Regarding the low average correlation coefficient, there are two factors that determine it, namely the motion artifacts and the low sampling rate. It is known that maxima are affected more seriously than minima by wave reflection phenomena. We conjecture that wave reflection phenomena may have produced some artifact(s) in our data. Conventionally, PTT measured between two consecutive minima has frequently been used as a surrogate for PTT. Thus, the correlation between OFF and EST_F appears to be higher than the correlation between OPP and EST_F. In theory, the correlation is highest between OFF and EST_F, but in practice, a low sampling rate produces an error. In our experiment, OFF was approximately 35 to 70 ms, calculated from two to four sample points. Fewer sample points may result in a higher error. Therefore, as shown in Table 2, we set the value of 0.6 as a performance threshold. In the cases where the correlation coefficient is higher than 0.6, we considered the correlation between results of our proposed method and the established one to be high.

The relationships among OFP, EST_P and EST_F are as follows:

(8) $$\left\{ \begin{array}{l} OFP=OFF+d_{p-f}\\ EST\_P=EST\_F+d_{p-f}\\ EST\_F=\alpha +\beta \times OFF \end{array}\right.$$

where the y-intercept α and the slope β are acquired from the linear regression between EST_P and OFP. $d_{p-f}$ is the systolic upstroke time, defined as the time interval between a maximum and a minimum in the fingertip signal. Thus, we can derive the following relationship between EST_P and OFP:

(9) $$EST\_P=\alpha -(\beta -1)\times d_{p-f}+\beta \times OFF$$

We assume that $d_{p-f}$ has little variability, and therefore EST_P and OFF have an approximately linear relationship:

(10) EST_P=k+β×OFF

where *k* is a constant of approximation.

As shown in Equation (10), EST_P and OFF have a linear relation. However, two errors impact the correlation between EST_P and OFP. First, an error is introduced because of the low sampling rate. Second, the degree of variability in $d_{p-f}$ also results in an error. As shown in Table 2, the experimental results demonstrated the highest correlation between EST_P and OFP. PTT is an important physiological parameter used in various experimental and clinical applications. Since the proposed method still produces an error, a better method for PPT measurement has yet to be found.

Currently, smartphones with two cameras are widely available. To our knowledge, however, irrespective of the type of the operating system, at the time of preparation of the present work, no smartphone allows simultaneous operation of the two cameras. Therefore, for our experiments we had to prepare custom hardware that used two cameras of type OV9715. The maximum resolution offered by this type of camera is 1280 × 800 pixels at a frame rate of 30 fps, which is lower than the one of the ordinary smartphone camera. In addition, the lens diameter of the typical smartphone camera is smaller than that of OV9715, and that determines lower optical shunting. Thus, we expect that the results of a real smartphones implementation will outperform those obtained in the present study. It is also a matter of minor design adjustment of the software from the side of manufacturers to allow simultaneous operation of the two smartphone cameras. Therefore, the proposed method for PTT measurement has a high potential for application in smartphones. 

### 5.2. Shortcomings and Improvements

Even though our proposed SFP algorithm showed a high-performance in detection of maxima and minima in PPGi signal with 98% SE and 97% PPV, and it also outperformed the Shin’s method used for comparison, it is also associated with an evident drawback. That is, the SFP algorithm is more time-consuming compared to other techniques because of the calculation of the proposed parabola. Therefore, to be implemented in the form of a smartphone application, the SFP algorithm needs to be further optimized.

## 6. Conclusions

Smartphones with two microcameras are very popular among the general public. In this study, we suggested an efficient method for estimation of PTT based on PPGi signals captured from two microcameras. We validated the method through comparisons with an established PTT estimation method. We found that the time intervals between the corresponding characteristic points of two subsequent waveforms in the PPGi signal have a high correlation with the corresponding intervals obtained through the established PTT measurement method. We suggest using the time interval OFP as the most reliable measure for PTT since it exhibits the best correlation coefficient (0.86 ± 0.06). The proposed PPGi method is suitable for implementation in smartphones. In the future, we will investigate the implementation of the proposed algorithm using mainstream smartphones. We will also investigate the impacts of different frame rates and spatial resolutions on the accuracy of PTT estimations to advance the widespread implementation of PTT estimation using smartphones.
